# Fully 3D-Printed Sampling-to-Detection Electrochemical Platform for Point-of-Care Measurement of Salivary Uric Acid

**DOI:** 10.1021/acsmeasuresciau.6c00046

**Published:** 2026-03-31

**Authors:** Ada Raucci, Wanda Cimmino, Cosimo Manna, Panagiota M. Kalligosfyri, Gennaro Persico, Michele Spinelli, Angela Amoresano, Waleed Alahmad, Stefano Cinti

**Affiliations:** † Department of Pharmacy, 9307 University of Naples Federico II, Via D. Montesano 49, Naples 80131, Italy; ‡ Department of Breast and Thoracic Oncology, Istituto Nazionale Tumori IRCCS Fondazione G. Pascale, Napoli 80131, Italy; § Department of Chemical Sciences, University of Naples Federico II, Naples 80126, Italy; ∥ Department of Chemistry, Faculty of Science, Chulalongkorn University, Bangkok 10330, Thailand; ⊥ Sbarro Institute for Cancer Research and Molecular Medicine, Center for Biotechnology, College of Science and Technology, Temple University, Philadelphia, Pennsylvania 19122, United States; # Bioelectronics Task Force at University of Naples Federico II, Via Cinthia 21, Naples 80126, Italy

**Keywords:** 3D-printing, additive manufacturing, electrochemical biosensor, uric acid, saliva, point-of-care testing

## Abstract

This research presents an innovative all-in-one 3D-printed electrochemical biosensor for noninvasive detection of uric acid in saliva. Uric acid is a key biomarker for monitoring disorders such as gout and kidney disease as well as assessing the progression of metabolic conditions. The 3D-printed platform integrates a microwell, saliva collection module, and a three-electrode system, including carbon black and Prussian-blue-modified electrodes with immobilized uricase, into a single compact device, streamlining the sampling-to-detection workflow. Initial optimization using screen-printed electrodes on polyester films with differential pulse voltammetry established baseline performance, while the fully integrated 3D-printed system, with the incorporation of uricase, achieved detection limits of 0.09 μM in phosphate-buffered saline and 0.1 μM in 20% diluted saliva. Comparative analysis with high-performance liquid chromatography confirmed the accuracy and reliability of the measurements. By combining nanomaterial-enhanced electrodes, enzymatic recognition, and additive manufacturing, this platform offers a portable, user-friendly, and low-cost solution for point-of-care uric acid monitoring. The integrated design minimizes sample handling, prevents contamination, and enables hygienic, noninvasive collection. This approach facilitates rapid diagnostic testing and real-time health monitoring, providing actionable information for personalized patient care. Overall, the fully 3D-printed biosensing system represents a significant advance in electrochemical biosensors, demonstrating the potential to improve the clinical management of uric acid-related conditions while enabling scalable, accessible, and point-of-care diagnostics.

## Graphical abstract

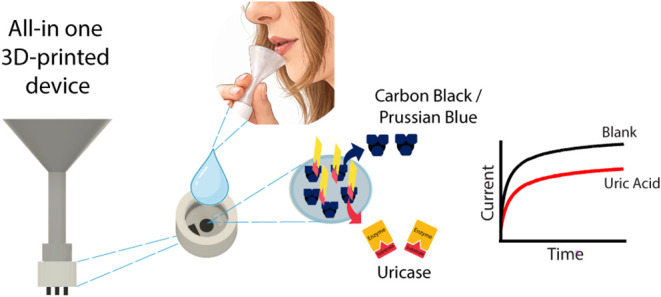

## Introduction

The concentrations of nutrients and metabolites in the blood are classic examples of the course of certain biological processes. Biofluids contain numerous nutrients and metabolites that can help to determine clinical risk, diagnosis, prognosis, and treatment efficacy. Biofluids are small molecules that play a significant role in physiological modulation. The constituents of biofluids are glucose, lipids, amino acids, and metabolites.^1^ Metabolic irregularities can lead to the disruption of metabolic processes, causing the accumulation or deficiency of metabolites, which are recognized as key features or biomarkers of various diseases. Patterns of metabolites that closely match an individual’s specific characteristics are valuable for predicting disease diagnosis, prognosis, and monitoring treatment progress.^2^ For example, the human body primarily produces acid, a compound with a heterocyclic structure, as a biomarker of metabolic syndrome during purine metabolism. Depending on the chemical environment within the body, it acts as a harmful antioxidant or pro-oxidant.^3^ In humans, the normal level of uric acid ranges from 6 mg dL^–1^ (360 μmol L^–1^) in women to 7 mg dL^–1^ (420 μmol L^–1^) in men.^4^ The level of acid in the body is commonly used as a signal for the diagnosis of various medical conditions. High levels of uric acid are associated with gout and osteoarthritis,^5^ hypertension, cardiovascular disease, and kidney disease; while lower levels of uric acid have been linked to conditions such as sclerosis, Parkinson’s disease, dementia, and optic nerve inflammation.^6,7^

Uric acid levels are some of the most important indicators of human health and are closely associated with various diseases. Understanding the significance of uric acid in these conditions underscores the importance of reliable detection for timely diagnosis and effective treatment strategies as well as continuous monitoring of disease progression. Based on clinical relevance, some of the spectral methods include spectrophotometry,^8^ colorimetry,^9,10^ chromatography,^11^ capillary electrophoresis,^12,13^ fluorescence^14,15^ and so on. The need for laboratory conditions, trained personnel, high-tech equipment, and specific reagents for signal detection limits these methods, despite their significant advances in clinical diagnostics.^16^ On the other hand, electrochemical methods have greater potential in uric acid determination due to their simplicity, short turnaround time, and selectivity.^17^ They reduce complexity in terms of equipment and specialized knowledge, making them suitable for real-time monitoring or use in clinical diagnostics.^18^ Electrochemical sensors are accessible and widely available in resourceful locations.^19^ These features indicate that electrochemical methods show potential for identifying uric acid levels.

The uricase enzyme helps the electrochemical sensor accurately measure acid levels by oxidizing uric acid in the presence of the uricase enzyme, which facilitates accurate measurement.^20^ Recent advances in the field of nanomaterials have significantly boosted the development of third-generation biosensor technology by improving the ability of biosensors to facilitate charge transfer between the biological recognition component and the electrode, which has led to a more efficient and accurate sensing process, making significant strides in biosensing applications. To achieve accuracy in sensors designed to detect substances such as uric acid, it is essential to strategically combine nanomaterials and enzymes to improve sensor sensitivity and selectivity. For example, carbon black (CB) and Prussian blue (PB) represent viable nanomaterials for surface modification of the electrode, which is to immobilize the uricase enzyme. CB enhances electron exchange between the analyte (uric acid) and the electrode, providing a better electrochemical response.^21^ PB acts as an electrocatalyst in the reduction of hydrogen peroxide (H_2_O_2_), a byproduct, in the reaction facilitated by uricase.^22^ There are numerous reports in the literature describing various applications of individual CBs or PBs with other materials and the immobilization of uricase enzyme for uric acid detection. Cruz et al. explored an electrochemical biosensor for uric acid detection, employing a graphite electrode modified with PB and uricase enzyme. It achieves a detection limit of 3.0 μM with good recovery from urine samples.^23^ In another study, Reanpan et al. developed a highly sensitive amperometric sensor using a CB-modified screen-printed carbon electrode and graphene oxide to effectively detect acid in the range of 0.05 to 2000 μM, achieving remarkable results.^24^ While highly performant, the implementation in a flow-injection amperometric configuration typically requires external fluidic handling and ancillary instrumentation, which can limit portability and user-friendliness for point-of-care salivary testing. Taken together, these studies not only demonstrate the value of PB-based mediation and CB-based conductivity enhancement but also highlight practical gaps in translating high analytical performance into an integrated, saliva-ready format. In parallel, advances in additive manufacturing and 3D-printing have opened new opportunities to address existing limitations in biosensor fabrication by enabling rapid prototyping, structural customization, and full system integration.^25^ The field has already demonstrated the feasibility of fully 3D-printed analytical platforms, including wearable ring-based sensors for sweat analysis,^26^ microwell systems for miRNA detection,^27^ and custom-designed trident-shaped sensors for ascorbic acid determination in fruits and vegetables.^28^ These examples highlight the growing importance of integrating sampling, sensing, and structural components into unified platforms.

Taking advantage of additive manufacturing, we developed a fully 3D-printed, all-in-one device that integrates the microwell, electrode ports, and saliva collection module into a single compact platform. 3D-printing enabled rapid prototyping, precise electrode positioning, fixed reaction volume, and easy customization of the geometry and electrode configuration. The screw-connected saliva collection module allows hygienic, noninvasive sampling without direct contact with the electrodes, while minimizing handling and reducing contamination risk. Conductive filaments allowed fabrication of functional electrodes directly within the same workflow, simplifying assembly and reducing production time. The detection system was initially optimized using a screen-printed electrode (SPE) on a polyester film in combination with differential pulse voltammetry (DPV). The sensor achieved a detection limit of 0.4 μM in a buffer solution and 0.7 μM in 1% diluted saliva samples. However, due to insufficient specificity in saliva, an enzyme biosensor was developed to overcome these limits and improve the detection performance. The biosensor, based on the enzyme uricase, achieved a significantly lower detection limit of 0.2 μM in 20% diluted real saliva samples. Building on this, the all-in-one 3D-printed device was developed and achieved a detection limit of 0.09 μM in buffer solution and 0.1 μM in 20% diluted saliva samples, demonstrating performance comparable to that of conventional SPE-based sensors. Immobilization of uricase on the working electrode enhanced the selectivity and sensitivity. This design integrates nanomaterial-enhanced electrodes, enzymatic recognition, and additive manufacturing into a single sampling-to-detection platform, providing a streamlined, reproducible, and user-friendly workflow for salivary uric acid measurement. Such integration addresses critical needs in clinical diagnostics, particularly for the monitoring and management of diseases such as gout and kidney disease. Overall, the 3D-printed, all-in-one biosensing platform represents a novel and practical approach to uric acid sensing with strong potential to advance electrochemical biosensors and improve patient care in uric acid-related conditions.

## Experimental Section

### Chemicals and Apparatus

All reagents used in this study are of analytical grade. Uric acid, PBS tablets (140 mM NaCl, 10 mM phosphate buffer, and 3 mM KCl) pH 7.2, hydrochloric acid, and uricase enzyme from *Candida sp.* recombinant, expressed in *E. coli*, were provided by Sigma-Aldrich. The dispersion of Prussian Blue [Fe_4_[Fe­(CN)_6_]] in Carbon Black was purchased from Cabot Corporation. We collected saliva samples from healthy volunteers. Conductive inks based on Ag/AgCl and graphite were provided by Sun Chemical. For the flexible substrate, we used Autostat HT5 (125 μm) from MacDermid, UK, which was chosen for its strength and flexibility characteristics, which are essential for our environmental applications. We used transparent adhesive tape, akin to packaging, to insulate the printed electrodes. Protopasta conductive PLA filament (1.75 mm; Protoplant, Vancouver, WA, USA), transparent R3D PLA filament (1.75 mm; 3DJake, Italy), and Basic White PLA filament (1.75 mm; Bambu Lab, Shenzen, China) were used for the 3D-printed device fabrication. The Bambu Lab A1 mini 3D-printer was sourced from Bambu Lab (Shenzhen, China). Autodesk Fusion 360 was employed for both device and electrode design, with Bambu Studio as the slicing software. All measurements were conducted using a portable potentiostat, EmStat3 (PalmSens, Netherlands), connected to a laptop.

### Screen-Printed Electrode Production

The SPEs were manually printed on a flexible polyester substrate by using specific conductive inks: Ag/AgCl for the reference electrode and graphite-based inks for the working and counter electrodes. The SPEs were consecutively treated in an oven at 100 °C for 30 min after each printing step. Commercial adhesive tape was used to insulate the printed electrodes. The working electrode had a diameter of 0.4 cm, and the electrochemical strips had dimensions of 2.5 cm × 1 cm (length x width).^29^

### All-in-One 3D-Printed Device Fabrication

The all-in-one device was fabricated entirely via fused filament fabrication (FFF) 3D-printing. It comprises a white 3D-printed microwell body as the electrochemical reaction chamber (∼800 μL volume), a screw-connected cylindrical saliva collection module with a cone-shaped distal end, and three interchangeable electrodes (working, counter, and reference; Figure S1). The microwell features three precisely aligned vertical ports for press-fit electrode integration and an internal threaded section for leak-free attachment of the saliva module. This allowed a click-in mechanism for easy device assembly. The cylindrical module includes a hollow chamber with a cone-shaped tip for contact-free saliva introduction, a distal enlarged section as a visual fill indicator, and a customized design to facilitate fluid mixing (Figure S2). All microwell and saliva collection components were fabricated using PLA filament via 3D-printing. PLA was selected due to its mechanical stability, ease of printing, and compatibility with aqueous biological samples. The working electrode (WE) and counter electrode (CE) were printed by using a conductive carbon-based filament suitable for electrochemical applications in aqueous media. The reference electrode (RE) consisted of a conductive 3D-printed base fabricated from the same conductive filament. The electrode surface was subsequently coated with a thin layer of silver/silver chloride (Ag/AgCl) paste following our group’s previously established protocol.^28^ The coated electrode was allowed to dry under ambient conditions to form a stable pseudoreference electrode suitable for electrochemical measurements. All 3D-printed electrodes were inserted via a click-in mechanism in the microwell. After their insertion, hot glue was used to close any gaps between the electrodes and the microwell, ensuring leak-proof performance. Additionally, all electrodes were designed with integrated conductive extensions terminating in pin connectors to enable direct electrical connection to a potentiostat (Figure S3).

### Biosensor Nanoengineering

For both SPEs and the 3D-printed working electrode the modification was performed by drop casting methodology. Four μL of a mixture composed of CB and PB, prepared by dissolving 1 mg of mixed powder in 1 mL of a DMF/H_2_O solution (1:1), were applied. This mixture was then dropped onto the working electrode area and allowed to dry. Following this, another 4 μL volume of the uricase enzyme at a concentration of 5 U/ml, optimized for the application, was added and also allowed to dry.^30^

### Uric Acid Quantification

We used differential pulse voltammetry (DPV) to optimize the experimental parameters and quantify uric acid. The experimental setup conditions for DPV were as follows: scan rate of 0.005 V/s, E step of 0.01 V, E-pulse of 0.2 V, and t pulse of 0.02 s. The DPV strategy is based on the direct oxidation of uric acid on the surface of a screen-printed carbon working electrode. No surface modifications were applied in this configuration. The voltammetric signal was acquired in the range 0.0–0.4 V, with the uric acid oxidation peak typically appearing at approximately +0.2 V vs Ag/AgCl. We made measurements using PBS at pH 7.2.

We added uricase enzyme to the electrode’s working area to increase the biosensor’s specificity and then used chronoamperometry for quantification. The level of uric acid is determined by measuring the current generated from the uricase reaction in the presence of uric acid, which is converted into hydrogen peroxide. Specifically, 100 μL of the sample was applied to the working area. A constant potential of 0 V (vs Ag/AgCl) was maintained for 60 s after a 2 min incubation period to facilitate the enzymatic reaction, during which uricase converts uric acid into an electroactive product, hydrogen peroxide. Notably, the production of hydrogen peroxide results in a decrease in current, as we observe a reduction reaction occurring at the electrode, as reported in Figure 1.

**Figure 1:**
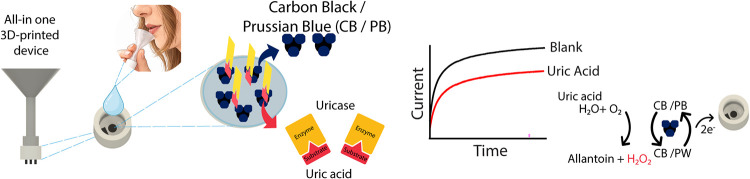
Schematic representation of the detection of uric acid in saliva by using the all-in-one 3D-printed electrochemical biosensor, nanoengineered with the conductivity enhancer (carbon black), the electrocatalyst (Prussian Blue), and the recognition bio element (uricase enzyme), and the typical chronoamperometric-based response at increasing concentration of uric acid.

## Results and Discussion

### Optimization of Experimental Parameters for Uric Acid Quantification

In this study, we optimized key experimental parameters to improve the detection efficiency of uric acid in solution using the DPV technique. The optimized parameters include scan rate, pulse time (T pulse), pulse voltage (E-pulse) applied to the electrochemical cell, and step potential, as shown in Figure S4, in the Supporting Info. These factors were carefully tuned to ensure optimal sensitivity and resolution in uric acid detection, which are crucial for clinical and diagnostic applications. Figure S4A shows the optimization of scan rate, where three settings were evaluated: 0.02, 0.01, and 0.005 V/s. The calibration curves reveal significant linear relationships between current and uric acid concentration for each scan rate. Among them, the scan rate of 0.005 V/s stood out for its excellent sensitivity and repeatability, making it an ideal choice for future measurements. This result suggests that slower scanning allows for better charge transfer and greater interaction between uric acid and the sensor, thus increasing the reliability of the readings. Next, in Figure S4B, we optimized the pulse T time, testing four values: 0.05, 0.02, 0.01, and 0.005 s, maintaining a scan rate of 0.005 V/s. The results show that a T pulse of 0.02 s offers the best repeatability, balancing stability and sensitivity. Although the values of 0.05 and 0.01 s showed higher sensitivity, they were less consistent. Consequently, the 0.02 s pulse was chosen for its reliable performance. In Figure S4C, we optimized the E-pulse, testing values of 0.3, 0.2, 0.1, and 0.05 V. Although a 0.3 V E-pulse provided the best linearity, its repeatability was lower. Thus, the 0.2 V pulse showed a good compromise, offering both sensitivity and stability, which are crucial aspects for discriminating between different concentrations of uric acid. Finally, Figure S4D shows the optimization of the E step parameter in DPV for uric acid detection, with settings fixed to a scan rate of 0.005 V/s, T pulse of 0.02 s, and E-pulse of 0.2 V. Three E step values were evaluated, with calibration curves showing the relationship between current and uric acid concentration. The E step of 0.01 V provided the best sensitivity, with a higher slope and repeatability, making it the optimal E step for further measurements.

### Calibration Curve for Uric Acid

After optimizing all experimental parameters, a calibration curve was obtained in PBS buffer at pH 7.2, in the range from 1 to 100 μM to determine the sensor response, as shown in Figure 2.

**Figure 2:**
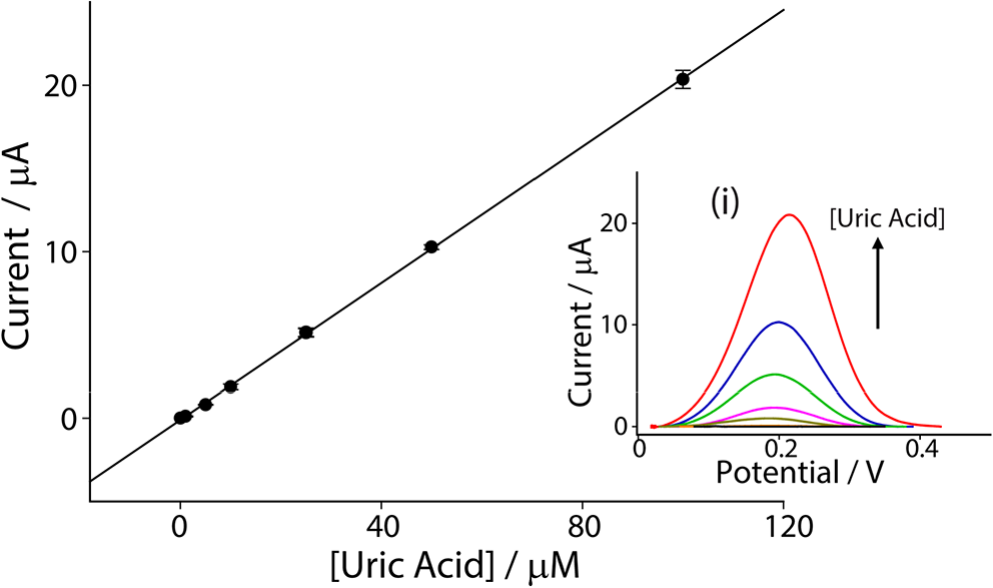
Calibration curve from 1 to 100 μM of uric acid in phosphate buffer, pH 7.2. Inset (i): recorded DPV in the absence and in the presence of 1, 5, 10, 25, 50, and 100 μM of uric acid. All experiments were performed in triplicate. Experimental parameters: E begin: 0.0 V, E end: 0.4 V, E step: 0.01 V, E-pulse: 0.3 V, t pulse: 0.02 s, Scan rate: 0.005 V/s.

The linear relationship between current and uric acid concentration is represented by the equation *y* = 0.205*x* – 0.109, *r* ^2^ = 0.99, indicating good sensitivity and linearity over the tested concentration range. The limit of detection (LOD) was estimated using eq 3σB/s, where σB is the standard deviation of the measurements and s is the slope of the calibration curve. We multiplied the LOD by a factor of 3.3 to determine the limit of quantification (LOQ). The LOD was determined to be 0.4 μM, and the LOQ was calculated to be 1.3 μM. These values suggest that the sensor is highly sensitive and capable of detecting low concentrations of uric acid in solution. The LOD and LOQ values suggest the sensor’s ability to detect uric acid in solution at different concentrations. In addition, a good repeatability of about 2% (calculated on five replicates) was obtained for this system.

### Optimization of the Saliva Matrix for Uric Acid Detection Using DPV

The analytical applicability and effectiveness of the final device were demonstrated by applying it to the determination of uric acid in saliva samples, as saliva-based sensors offer a noninvasive alternative to blood tests for monitoring uric acid levels.^31^ First, the impact of salivary matrix dilution on uric acid detection using the DPV technique was studied. Figure 3 presents the current response for different saliva dilutions: 1, 20, 50, and 100%. The study tested different saliva dilutions to minimize the matrix effect, which could distort measurements due to the complex biological composition of saliva. The results show that as the saliva concentration decreases to 1%, higher sensitivity and reproducibility are observed. This indicates that higher dilutions reduce the interference of the salivary matrix, making it better suited to detecting uric acid reliably. In particular, 1% saliva is essentially the only practically useful dilution for the nonenzymatic DPV sensor: at this dilution, uric acid can still be detected with high sensitivity and a well-defined linear trend, while matrix effects are sufficiently suppressed to guarantee good repeatability. At higher saliva contents (20, 50, and 100%), the increasing amount of proteins, salts, and other electroactive species progressively lowers the slope and worsens the linear correlation, so these conditions are not suitable for robust quantitative DPV, even though calibration lines can still be fitted. For the 20% saliva dilution, the calibration is described by the equation is *y* = 0.213*x* + 5553 with a sensitivity (slope) of 0.213, *r* ^2^ = 0.718, a LOD of 3.8 μM and a LOQ of 12.6 μM, confirming the inferior analytical performance compared to 1% saliva and supporting the choice of 1% as the preferred working dilution for the DPV strategy.

**Figure 3:**
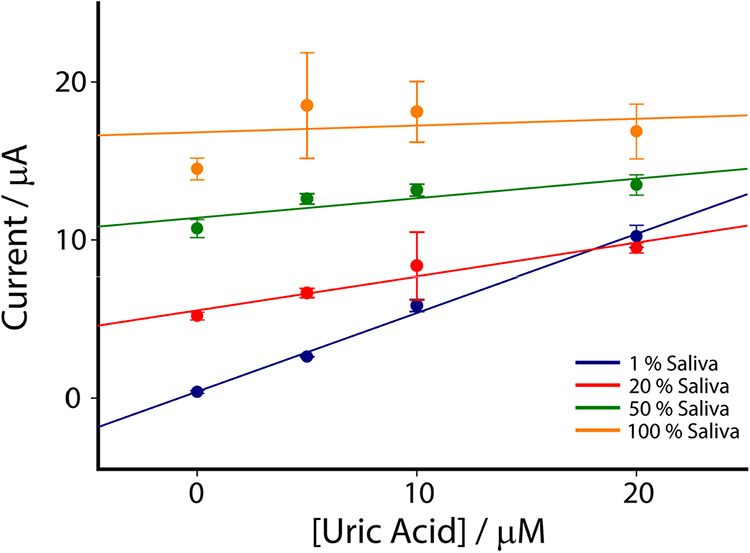
Study of the saliva- matrix effect. Calibration curves in the range between 0 and 20 μM of uric acid in different saliva dilutions: saliva diluted to 1% (blue), to 20% (red), 50% (green), and 100% (orange). All experiments were performed in triplicate, and the experimental conditions are as shown in the caption of Figure 2.

In Figure 3, the linearity of a calibration curve for uric acid in 1% saliva is shown, plotting the current versus uric acid concentration from 5 to 20 μM. The linearity of the curve with equation *y* = 0.4983 + 0.4140, *r* ^2^ = 0.9845 demonstrates good sensitivity and reproducibility in this dilute matrix. The LOD obtained was 0.7 μM with an LOQ of 2.3 μM. Under these conditions, we also evaluated the effect of typical electroactive salivary components on the DPV response in 1% saliva by testing ascorbic acid, dopamine, paracetamol, and lactic acid, each at 10 μM (Figure S5A, SI). Dopamine, paracetamol, and ascorbic acid produced smaller oxidation peaks within the uric acid potential region, confirming a partial overlap of their electroactivity, whereas lactic acid generated only a negligible signal. The uric acid peak nonetheless remained the most intense feature of the voltammogram, allowing reliable quantification in diluted saliva, as supported by the calibration curve in Figure 3.

### Optimization of the Enzymatic Biosensor for Uric Acid Detection Using Chronoamperometry

In order to increase the specificity of the sensor for uric acid, we finally developed a biosensor based on the uricase enzyme combined with CB and PB nanomaterials for sensor modification. Therefore, as a first step, the experimental setup of the biosensor was optimized in order to achieve satisfactory sensitivity and repeatability, as shown in Figure 4.

**Figure 4:**
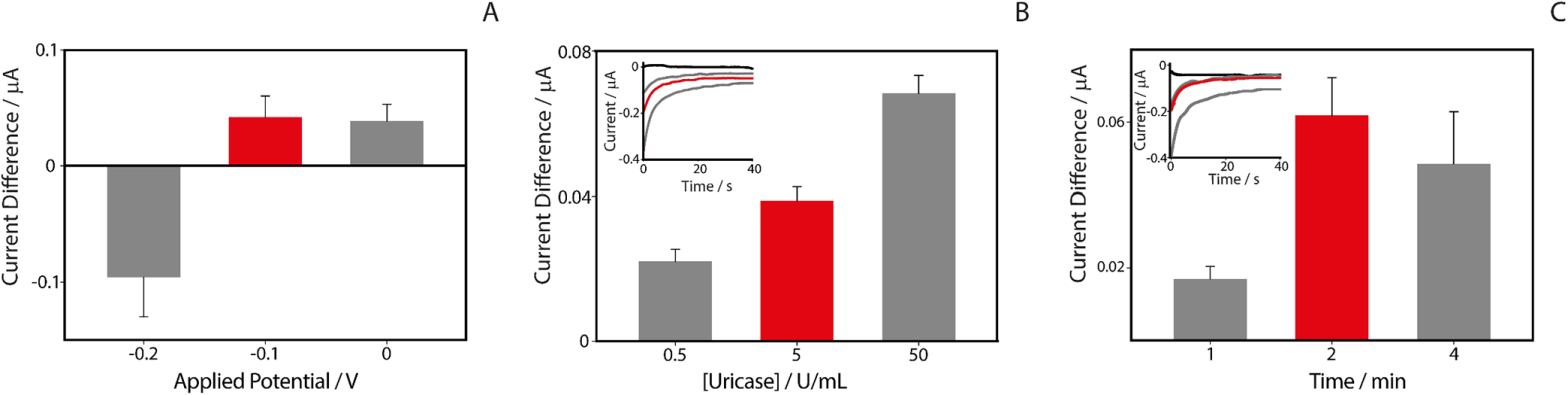
(A) Optimization of the constant potential applied during the chronoamperometry, between −0.2, −0.1, and 0 V; (B) evaluation of the enzymatic activity between 0.5 and 5 and 50 U/mL uricase in the presence of 10 μM uric acid, with an applied potential of −0.1 V (versus Ag/AgCl); and (C) optimization of the enzymatic reaction waiting time between 1, 2, and 4 min. All experiments were performed in triplicate and were performed in PBS buffer at pH 7.2.

The figure illustrates the optimization process for creating an enzymatic biosensor for uric acid detection using chronoamperometry. Key parameters, such as applied potential, enzyme concentration, and reaction time, were considered. The study in Figure 4A shows the sensitivity achieved by applying different potentials (−0.2, −0.1, and 0.0 V) during chronoamperometric measurements. The results show that −0.2 V leads to a decrease in the signal, suggesting interference. A potential of −0.1 V (vs Ag/AgCl) was chosen for further study because of the stable and positive signal change. Figure 4B shows the impact of the uricase enzyme concentration on signal variation and reaction time. Three concentrations were tested: 0.5, 5, and 50 U/mL. The concentration of 5 U/mL showed significant signal variation with good repeatability, making it the optimal concentration for biosensor modification. Figure 4C evaluates the impact of reaction time on the signal, comparing intervals of 1, 2, and 4 min. A 2 min reaction time significantly improved signal sensitivity, while longer times (4 min) did not. A 2 min waiting time provided the best compromise between allowing the enzyme to generate sufficient hydrogen peroxide and keeping the response clearly dependent on uric acid concentration. After only 1 min, the reaction is not advanced enough, so the current is lower and less sensitive to concentration changes, whereas at 4 min, uric acid near the enzyme layer is partly consumed and products accumulate, making the system less “fresh” and reducing the increase in current with concentration. For this reason, 2 min was selected as the optimal reaction time, ensuring the highest and most reproducible sensitivity across the tested uric acid range.

### Analytical Performance

Subsequently to the optimization of amperometric experimental parameters, the biosensor was tested for uric acid determination in the range 0–100 μM in phosphate buffer solution, as shown in Figure 5. The current values used for constructing the calibration curves were measured at 60 s after potential application, a time point chosen based on stabilization of the amperometric signal across all concentrations. We first evaluated the performance of the uricase-based biosensor on SPEs for uric acid determination in the 0–100 μM range in phosphate buffer (Figure 5A).

**Figure 5:**
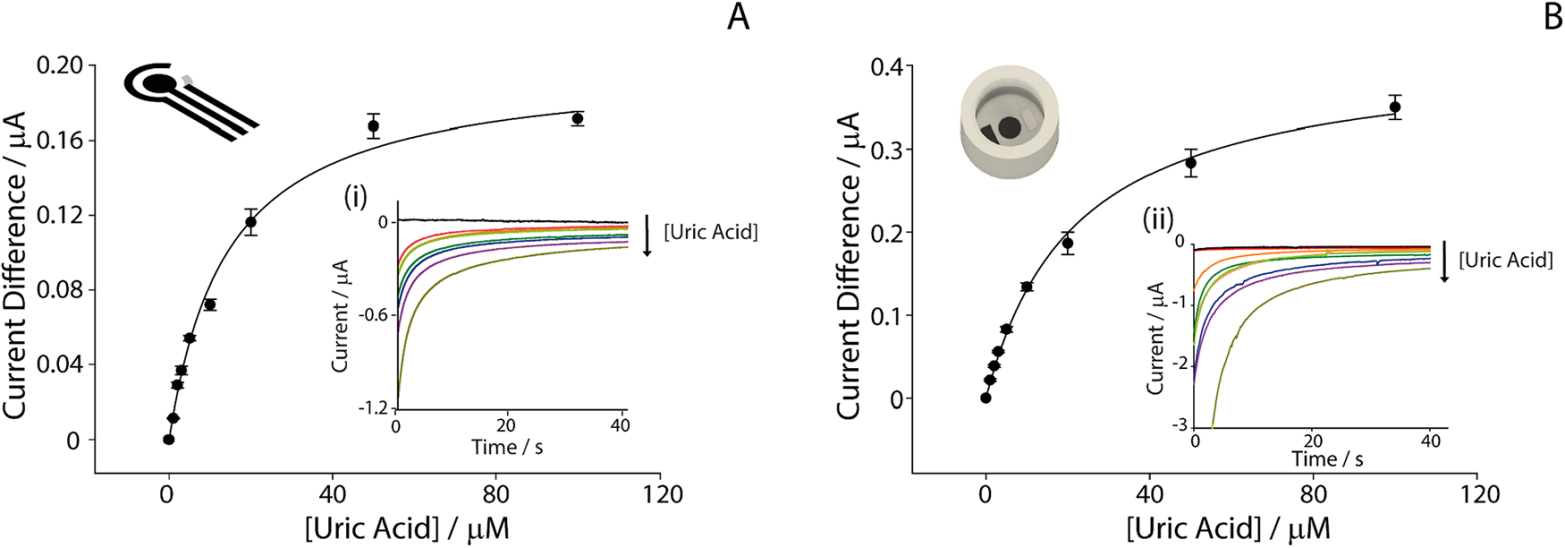
Calibration curve in phosphate buffer solution, obtained in the range between 0 and 100 μM uric acid using (A) the uricase-based biosensor with SPEs and (B) the uricase-based biosensor with 3D-printed electrodes. Insets (i) and (ii): corresponding chronoamperometric recordings obtained in the range between 0 and 100 μM of uric acid. Experimental conditions are the same as described in previous optimization studies. All of the measurements were made in triplicate.

The steady-state current at 60 s was fitted to *I = I*_max_ *· C*/(*K*_*m*_ *+ C)*, yielding *I*_max_ = 0.200 μA, *K*_m_ = 14.2 μM, and *r* ^2^ = 0.988, confirming classical Michaelis–Menten saturation behavior. Linearity was obtained between 0 and 5 μM (Figure S6A, SI), with *y* = 0.0104*x* + 0.004, and *r* ^2^ = 0.971. Each point on the curve was obtained from three replicates made with different electrodes. The LOD was found to be about 0.1 μM, while the LOQ was set at 0.3 μM, with a relative standard deviation calculated from five replicates of 6%.

We then extended this configuration to a 3D all-in-one electrode architecture specifically designed to combine sample collection and electrochemical readout on the same support so that a single integrated device can directly receive the saliva droplet and perform amperometric detection without additional transfer or handling steps. Under the same conditions in phosphate buffer, the 3D-printed biosensor displayed Michaelis–Menten-type behavior over 0–100 μM (Figure 5B), with *I*_max_ = 0,416 μA, *K*_m_ = 21.79 μM, and *r* ^2^ = 0.990; its linear range (Figure S6B) extended from 0 to 10 μM and was described by the equation *y* = 0.0131*x* + 0.008 with *r* ^2^ = 0.980. The LOD obtained was 0.09 μM, with an LOQ of 0.3 μM, and a relative standard deviation of 4%. Taken together, these results indicate that the 3D all-in-one platform not only simplifies sampling and measurement but also provides higher maximum current, improved low-concentration performance, and better precision compared to planar SPEs.

To evaluate the application of the biosensor, the applicability was first tested using real saliva samples. In particular, we studied the saliva matrix effect with the uricase-based biosensor strategy by spiking uric acid (0–5 μM) into saliva diluted to 100, 50, and 20%, as shown in Figure S7. These experiments revealed that a 20% saliva dilution offered the best compromise between signal intensity, linearity, and repeatability: at this dilution, the enzymatic current remained sufficiently high, while matrix-related interferences and viscosity effects were attenuated, resulting in amperometric responses that were not only more reproducible but also larger in magnitude than those obtained in 50% and 100% saliva. On this basis, subsequent calibrations in saliva were carried out at 20% dilution for both the SPE-based biosensor and the 3D all-in-one platform (Figure 6), enabling reliable quantification of uric acid in clinically relevant salivary levels.

**Figure 6:**
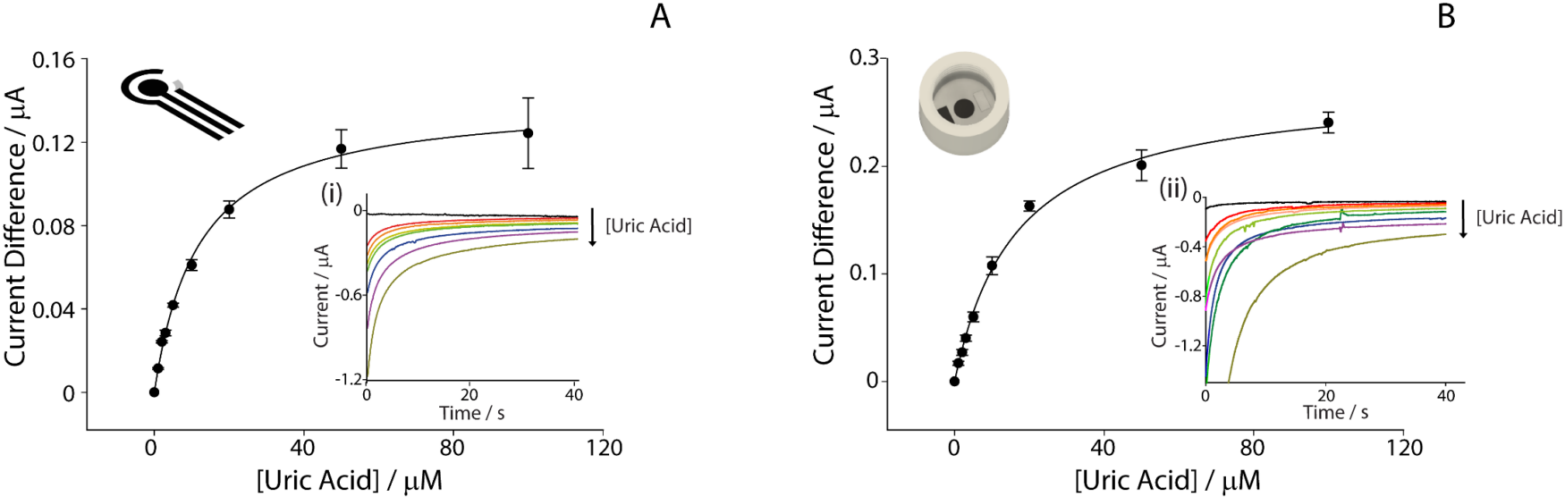
Calibration curve in 20% saliva, obtained in the range between 0 and 100 μM uric acid using (A) the uricase-based biosensor with SPEs and (B) the uricase-based biosensor with 3D-printed electrodes. Insets (i) and (ii): corresponding chronoamperometric recordings obtained in the range between 0 and 100 μM of uric acid. Experimental conditions are the same as described in previous optimization studies. All the measurements were made in triplicate.

As shown in Figure 6A, for the SPE-based biosensor, uric acid concentrations between 1 and 100 μM were tested, with the main plot displaying a proportional increase in current as the concentration rises and inset (i) showing the corresponding chronoamperometric traces. Fitting the calibration data in 20% saliva to the Michaelis–Menten equation yielded *I*_max_ = 0.140 μA, *K*_m_ = 11.7 μM, and *r* ^2^ = 0.978; the linear range from 0 to 5 μM (Figure S8A) was described by *y* = 0.007*x* + 0.005 with an *r* ^2^ value of 0.965, giving a LOD of 0.2 μM, an LOQ of 0.6 μM, and a repeatability of about 7% (*n* = 5). Compared with the previously optimized nonenzymatic sensor, this enzymatic configuration provided higher specificity and a significantly lower detection limit in saliva. For the 3D all-in-one biosensor, the calibration in 20% saliva (Figure 6B) also followed Michaelis–Menten behavior, with *I*_max_ = 0.275 μA, *K*_m_ = 16.36 μM and *r* ^2^ = 0.988, indicating efficient enzyme–substrate interaction on the 3D-printed architecture and a higher maximum current than the planar SPE configuration. The corresponding linear range (Figure S8B) extended from 0 to 10 μM and was fitted by *y* = 0.010*x* + 0.006 with *r* ^2^ = 0.981, yielding an LOD of 0.1 μM, an LOQ of 0.33 μM, and an RSD of 6% over five replicates. Notably, in the 3D all-in-one format, the sampling and 5-fold dilution of saliva to 20% are achieved in a single step directly on the device, so that the user only needs to deposit the raw saliva droplet while the integrated architecture simultaneously performs on-chip dilution and amperometric detection, further supporting its suitability for practical point-of-care use. Clinical studies indicate that salivary uric acid in healthy adults typically lies in the low- to midmicromolar range, with reported mean values around 90–240 μM (1.5–4 mg/dL) and reference intervals roughly between 0.3 and 6.1 mg/dL (18–360 μM).^32^ In this framework, the 0–5 and 0–10 μM linear ranges obtained in 20% saliva (corresponding to 0–25 and 0–50 μM in undiluted saliva), together with the full calibrated 0–100 μM interval, are well aligned with the clinically relevant salivary uric acid window and allow reliable quantification from physiological up to clearly elevated levels.

Moreover, we evaluated the effect of common salivary electroactive species under the operating conditions of a uricase-based amperometric biosensor. Interference tests were performed in 20% saliva using the same incubation and chronoamperometric protocol adopted for the calibration plots. In this configuration, the combination of the selective uricase reaction (conversion of uric acid into H_2_O_2_) with Prussian Blue-mediated detection at low applied potential resulted in only minor current variations in the presence of ascorbic acid, dopamine, paracetamol, and lactic acid at physiologically relevant levels, with changes remaining within the experimental variability of the uric acid signal. These results, reported in Figure S4B, support the enhanced specificity of the enzymatic amperometric strategy in saliva and complement the selectivity study carried out for the DPV approach.

Additionally, HPLC experiments, as the gold-standard method for uric acid quantification, were carried out to evaluate the performance of the biosensor on real saliva samples. The paired biosensor–HPLC results are summarized in a correlation plot (Figure S9, SI), which shows a Pearson correlation coefficient *r* = 0.9809 and an adjusted coefficient of determination *R* ^2^ = 0.897, indicating that almost 90% of the variability in the HPLC concentrations is explained by the biosensor response. The corresponding regression line exhibits a slope of 1.54 ± 0.26 and an intercept of −231 ± 118 μM, revealing a systematic proportional bias but confirming that the biosensor closely follows the trend of the reference method over the investigated range. To better assess agreement, a Bland–Altman analysis was also performed: the mean bias was −9.8 μM (≈2% relative deviation), with limits of agreement of −157.5 to 137.9 μM, indicating no relevant systematic error and an acceptable variability for enzymatic electrochemical detection over the wide salivary uric acid range considered. Overall, the combined regression and Bland–Altman results support the use of the device as a practical tool for semiquantitative detection and point-of-care monitoring of salivary uric acid, complementing conventional HPLC analysis. Taken together, these data support the use of the device as a practical tool for semiquantitative detection and monitoring of uric acid in real saliva samples, complementing conventional HPLC analysis in point-of-care settings.

## Conclusions

Accurate monitoring of uric acid is essential for the diagnosis and management of health conditions, such as gout, kidney disease, and metabolic disorders. In this study, we developed a fully integrated, all-in-one 3D-printed electrochemical (bio)­sensor for noninvasive uric acid detection in saliva, using this biomarker as a proof of concept to demonstrate the versatility of the platform. Beyond uric acid, the device architecture enables straightforward immobilization of other biomolecules including enzymes, antibodies, or aptamers, allowing adaptation to a wide range of clinically relevant targets. Thus, the proposed 3D-printed system represents not only a saliva-based uric acid sensor but also a flexible platform technology for noninvasive monitoring of multiple diseases at the point of care.

Under optimized conditions, the sensor exhibited a sensitive calibration curve in buffer, with a detection limit of 0.4 μM and a quantification limit of 1 μM. Saliva analysis indicated that a 1% dilution provided optimal performance, achieving a detection limit of 0.7 μM. Biosensor performance was further enhanced through the incorporation of uricase together with a CB/PB dispersion and optimization of chronoamperometric parameters, including applied potential, reaction time, and enzyme loading. The optimized biosensing chemistry was subsequently translated into the fully 3D-printed device, which integrates a microwell reaction chamber, a screw-connected saliva collection module, and a three-electrode system within a single compact structure. This configuration achieved detection limits of approximately 0.09 μM in the buffer and 0.1 μM in saliva.

The 3D-printed architecture offers several advantages over conventional planar strip geometries: it integrates sampling, saliva handling, and detection into one device, minimizing sample manipulation and contamination risk; enables hygienic, contact-free saliva collection; ensures precise electrode positioning and fixed reaction volumes for improved reproducibility; and allows direct fabrication of functional electrodes within the same additive manufacturing workflow. The customizable, portable, and user-friendly design supports scalable, low-cost production suitable for point-of-care deployment. Validation against HPLC confirmed the analytical accuracy and reliability. Overall, the integrated 3D platform represents a practical and versatile solution for noninvasive biomarker monitoring.

## Supplementary Material



## Notes

Assembly and the dimensions of the all-in-one 3D-printed device (Figure S1 and S2), photos of the real system configuration (Figure S3) optimization of the proposed sensing system (Figure S4), interference studies (Figure S5), linear ranges of the uricase-based biosensors in PBS (Figure S6), study of the saliva matrix effect with the uricase-based biosensor strategy (Figure S7), linear range of the uricase-based biosensors in saliva (Figure S8), and correlation with the gold-standard method for uric acid quantification (Figure S9) (PDF)
