# Supplementary material for: Fully 3D-Printed Sampling-to-Detection Electrochemical Platform for Point-of-Care Measurement of Salivary Uric Acid
Source: ACS Meas Sci Au. 2026 Mar 31;6(3):775–83. doi: 10.1021/acsmeasuresciau.6c00046 (PMC13281170; doi:10.1021/acsmeasuresciau.6c00046)
Supplement: Supplementary file 1 [file tg6c00046_si_001.pdf]

A fully 3D-printed sampling-to-detection electrochemical platform for point-of-care measurement of salivary uric acid

Ada Raucci[1,2,*], Wanda Cimmino[1], Cosimo Manna[1], Panagiota M. Kalligosfyri[1], Gennaro Persico[1], Michele Spinelli,[3] Angela Amoresano,[3] Waleed Alahmad[4], and Stefano Cinti[1,4,5,6*]

[1] Department of Pharmacy, University of Naples "Federico II", Via D. Montesano 49, Naples 80131, Italy.

[2] Department of Breast and Thoracic Oncology, Istituto Nazionale Tumori IRCCS Fondazione G. Pascale, Napoli 80131, Italy

[3] Department of Chemical Sciences, University of Naples "Federico II", Naples 80126, Italy.

[4] Department of Chemistry, Faculty of Science, Chulalongkorn University, Bangkok, 10330, Thailand.

[5] Sbarro Institute for Cancer Research and Molecular Medicine, Center for Biotechnology, College of Science and Technology, Temple University, Philadelphia, Pennsylvania 19122, United States

[6] Bioelectronics Task Force at University of Naples Federico II, Via Cinthia 21, Naples 80126, Italy.

[*] Corresponding Authors

E-mail Addresses: ada.raucci@unina.it; stefano.cinti@unina.it

Table of contents

Figure S1. Assembly and dimensions of the fully 3D printed device**.** Page S2

Figure S2. Assembly and dimensions of the fully 3D printed device**.** Page S2

Figures S3. Photos with the real system configuration. Page S3

Figure S4. Optimization of the experimental parameters. Page S4

Figure S5. Interferents study . Page S5

Figure S6. Linear ranges of the uricase-based biosensors in PBS. Page S5

Figure S7. Study of the saliva- matrix effect with the uricase-based biosensor strategy. Page S6

Figure S8. Linear ranges of the uricase-based biosensors in saliva. Page S6

Figure S9. Correlation with HPLC. Page S7

Assembly and dimensions of the fully 3D-printed integrated device

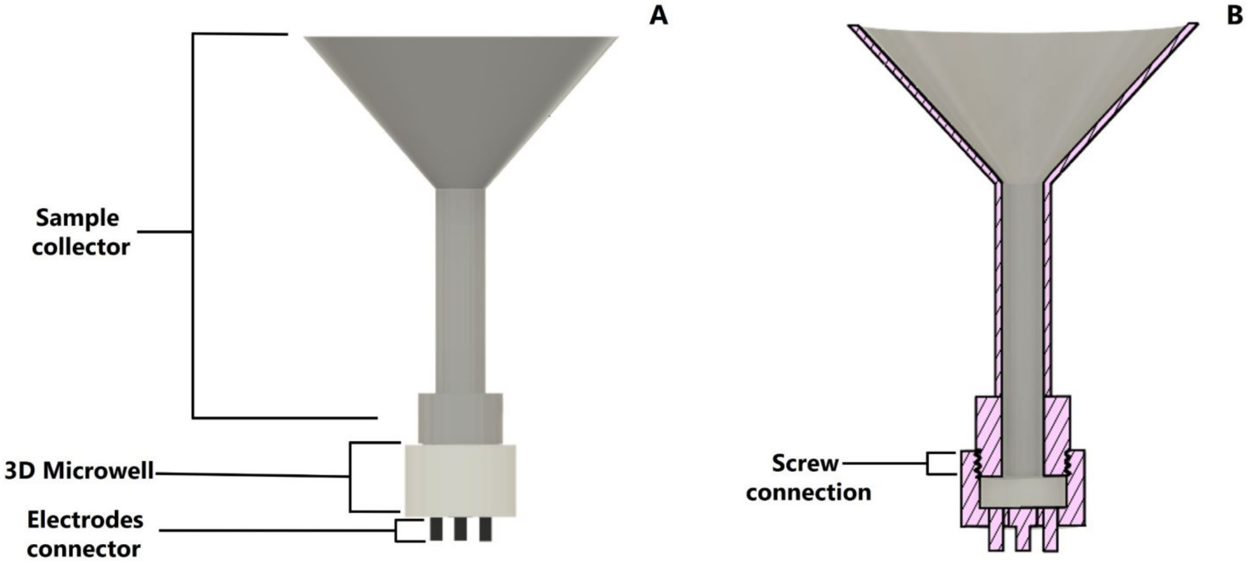

Figure S1. The all-in-one 3D-printed platform consists of: A) three electrodes with integrated connectors for direct connection to the potentiostat; a microwell serving as the electrochemical reaction chamber for liquid handling; and a sample collection module B) attached via a screw connection, enabling seamless integration of saliva sampling and electrochemical detection within a single compact device.

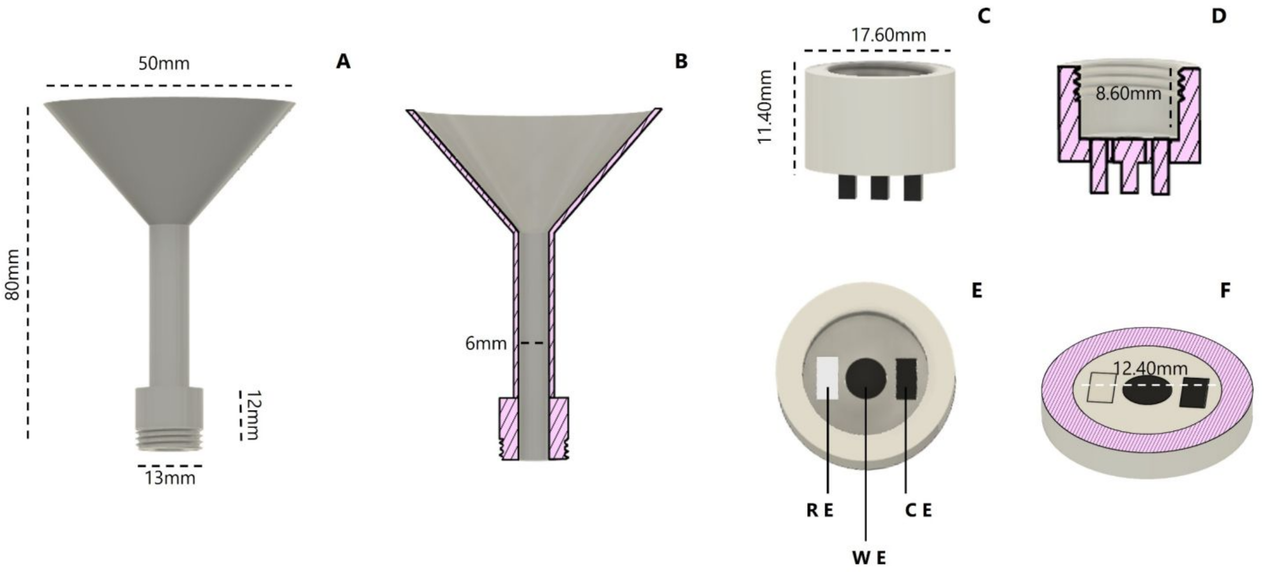

Figure S2. The dimensions of A) B) the cone-shaped sample collector and C), D), E), F)
the 3D microwell with the click-in mechanism for the assembly of the 3D-printed electrodes.

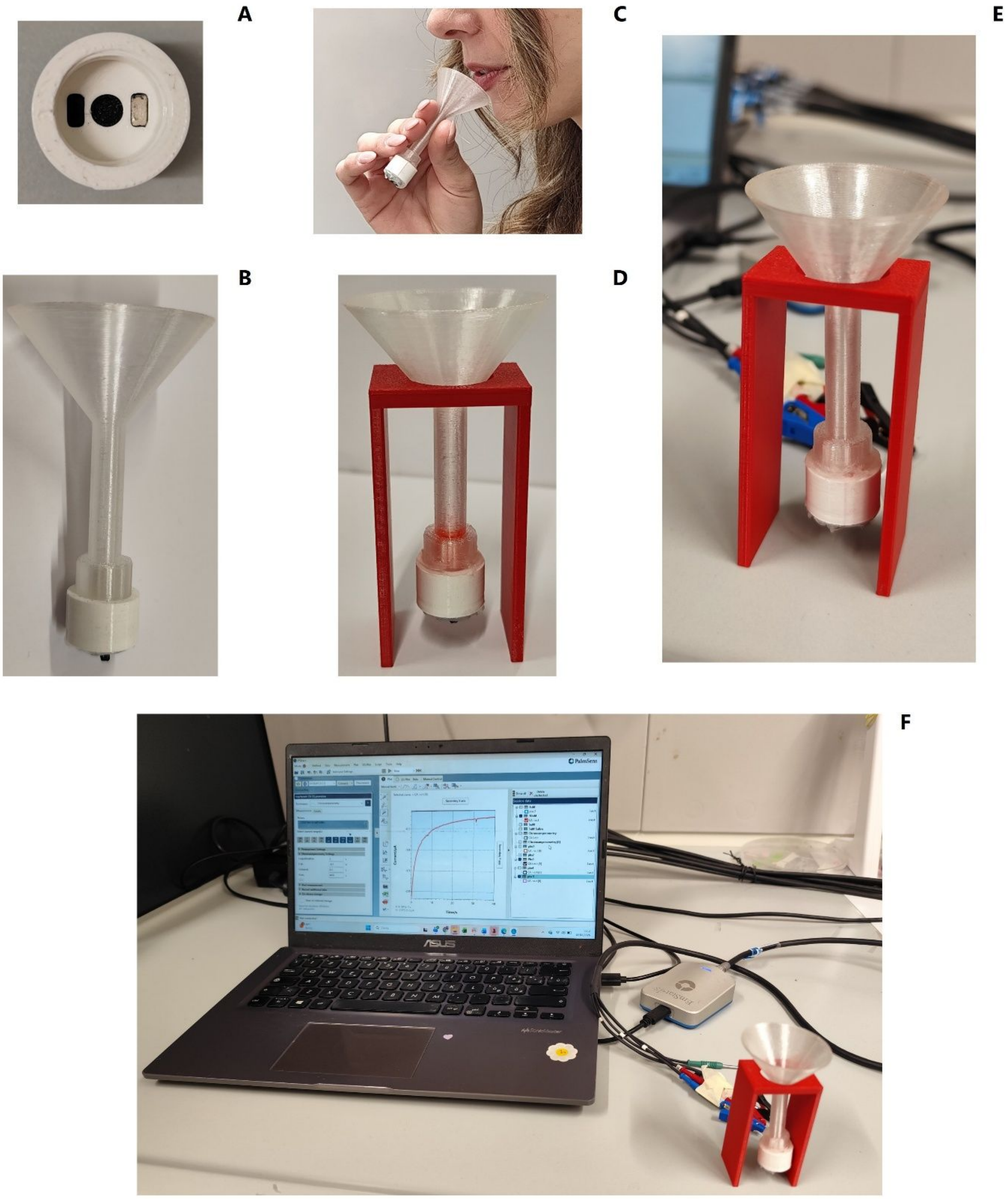

Figure S3. A) The microwell assembled using a click-in mechanism with the 3D-printed electrodes. B) The saliva collection module assembled via a screw connection to the 3D-printed electrochemical microwell. C) A user providing a saliva sample directly into the preloaded microwell containing 800 μL of buffer to achieve a five-fold dilution. D) The device containing the diluted sample (red dye was used solely to visualize the height of the final solution volume) positioned in a 3D-printed designated stand for measurement. E) The all-in-one 3D-printed platform connected to the potentiostat. F) The complete system configuration, from sample collection to electrochemical measurement.

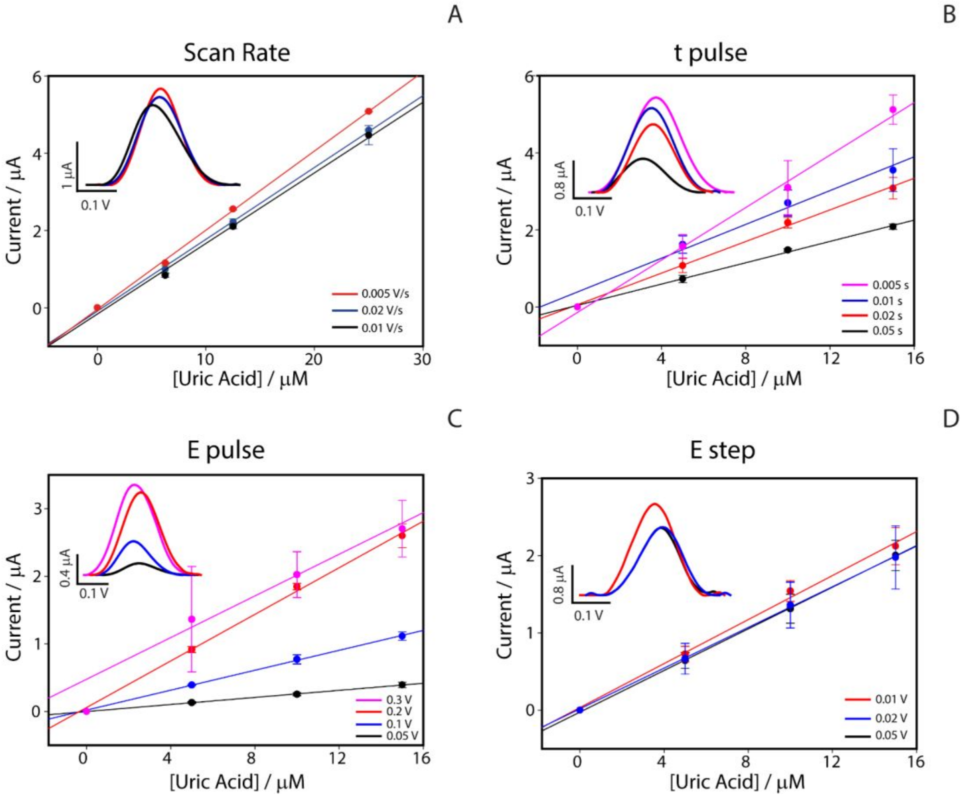

Figure S4. Optimization of experimental parameters using the DPV technique. A) Scan Rate Optimization: Calibration curves obtained at three different scan rates: 0.01 V/s (black), 0.02 V/s (blue), and 0.05 V/s (red); B) Pulse Time Optimization (t pulse): Calibration curves for varying pulse times: 0.05 s (black), 0.02 s (red), 0.01 s (blue), and 0.005 s (pink); C) Pulse Potential Optimization (E pulse): Calibration curves at different pulse potentials: 0.05 V (black), 0.1 V (blue), 0.2 V (red), and 0.3 V (green); D) Step Potential Optimization (E step): Calibration curves with varying step potentials: 0.05 V (black), 0.02 V (blue), and 0.01 V (red); Each curve illustrates the relationship between current response and experimental parameters, highlighting optimal conditions for accurate detection of uric acid. All measurements were performed in phosphate buffer at pH 7.2.

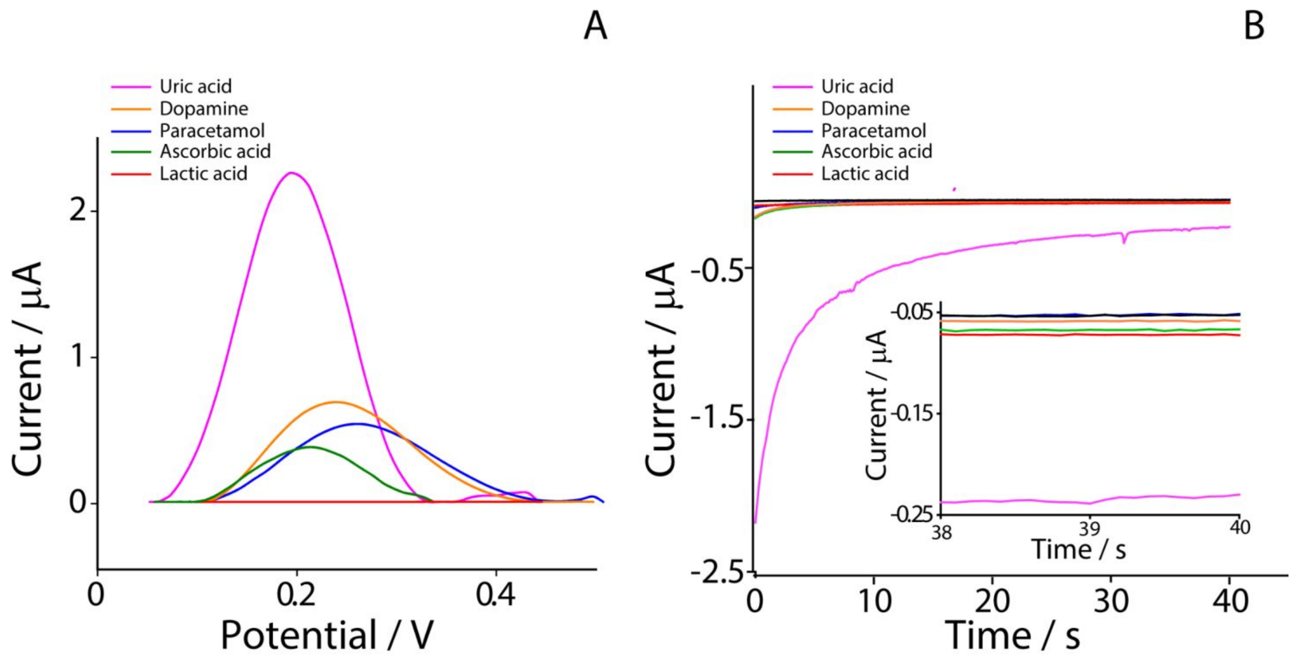

Figure S5. Study of interferents: measurements in the presence of uric acid (pink), dopamine (orange), paracetamol (blue), ascorbic acid (green) and lactic acid (red), at a concentration of 10 μM in saliva, using: A) the DPV strategy in 1% saliva and B) the uricase based amperometric biosensor in 20% saliva.

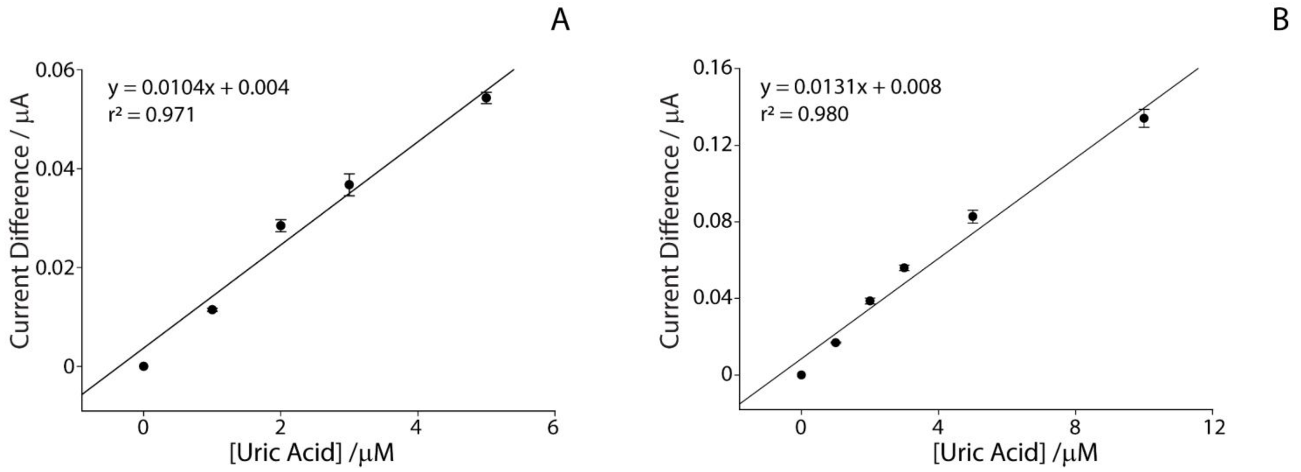

Figure S6. Regression line corresponding to the linear range of the calibration curves for uric acid in phosphate buffer. A) Current difference (μA) vs uric acid concentration (μM) in PBS using the uricase-based biosensor with SPEs, with the regression line in the 0-5 μM range and the corresponding linear equation. B) Current difference (μA) vs uric acid concentration (μM) in PBS using the uricase-based biosensor with 3D electrodes, with the regression line in the 0-10 μM range and the corresponding linear equation.

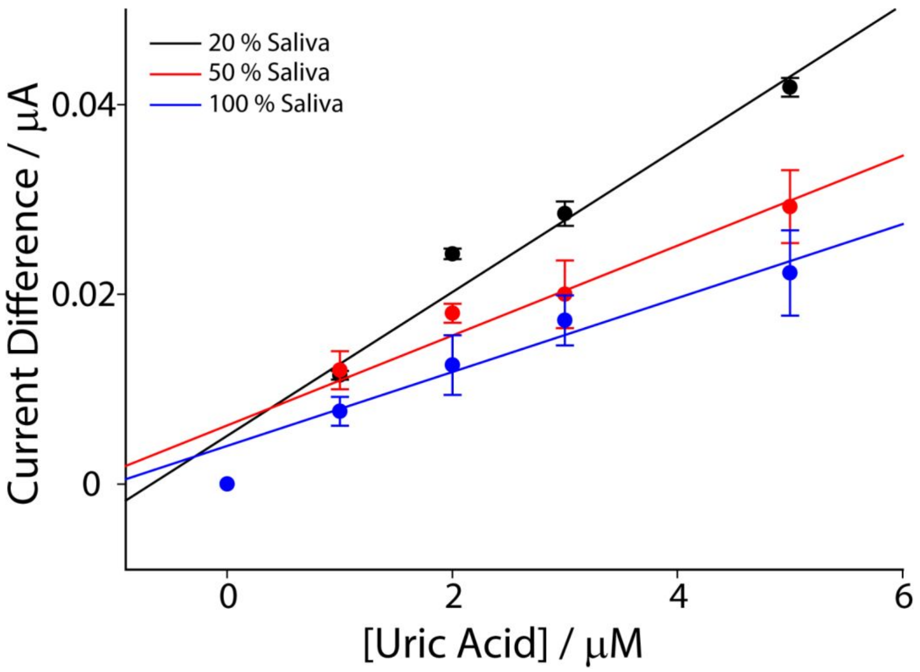

Figure S7. Study of the saliva- matrix effect with the uricase-based biosensor strategy. Calibration curves in the range between 0 and 5 mM of uric acid in different saliva dilutions: saliva diluted to 20% (black), to 50% (red) and 100% (blue). All experiments were performed in triplicate.

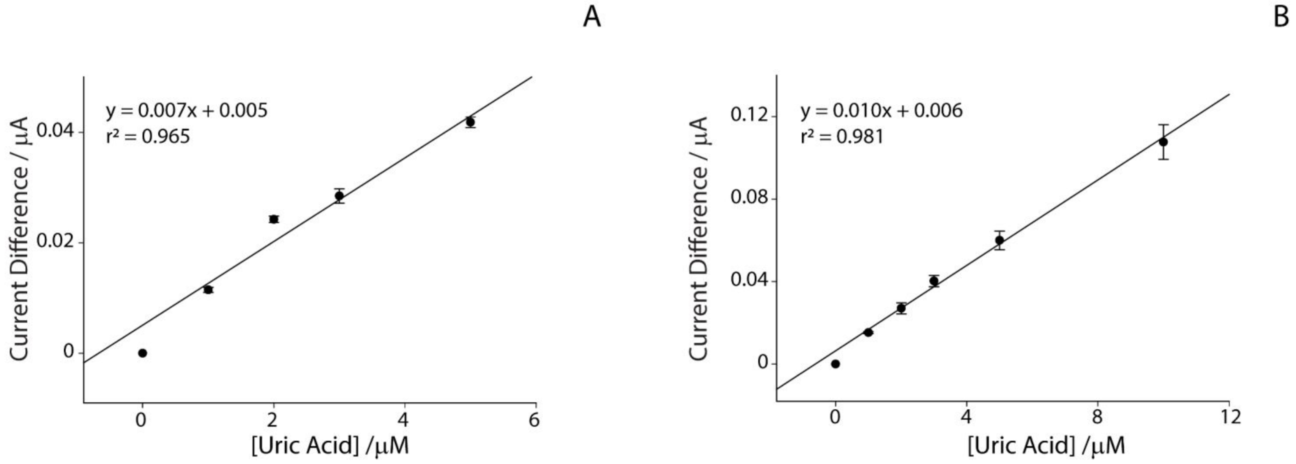

Figure S8. Regression line corresponding to the linear range of the calibration curves for uric acid in 20% saliva. A) Current difference (µA) vs uric acid concentration (µM) in saliva using the uricase-based biosensor with SPEs, with the regression line in the 0-5 µM range and the corresponding linear equation. B) Current difference (µA) vs uric acid concentration (µM) in saliva using the uricase-based biosensor with 3D electrodes, with the regression line in the 0-10 µM range and the corresponding linear equation.

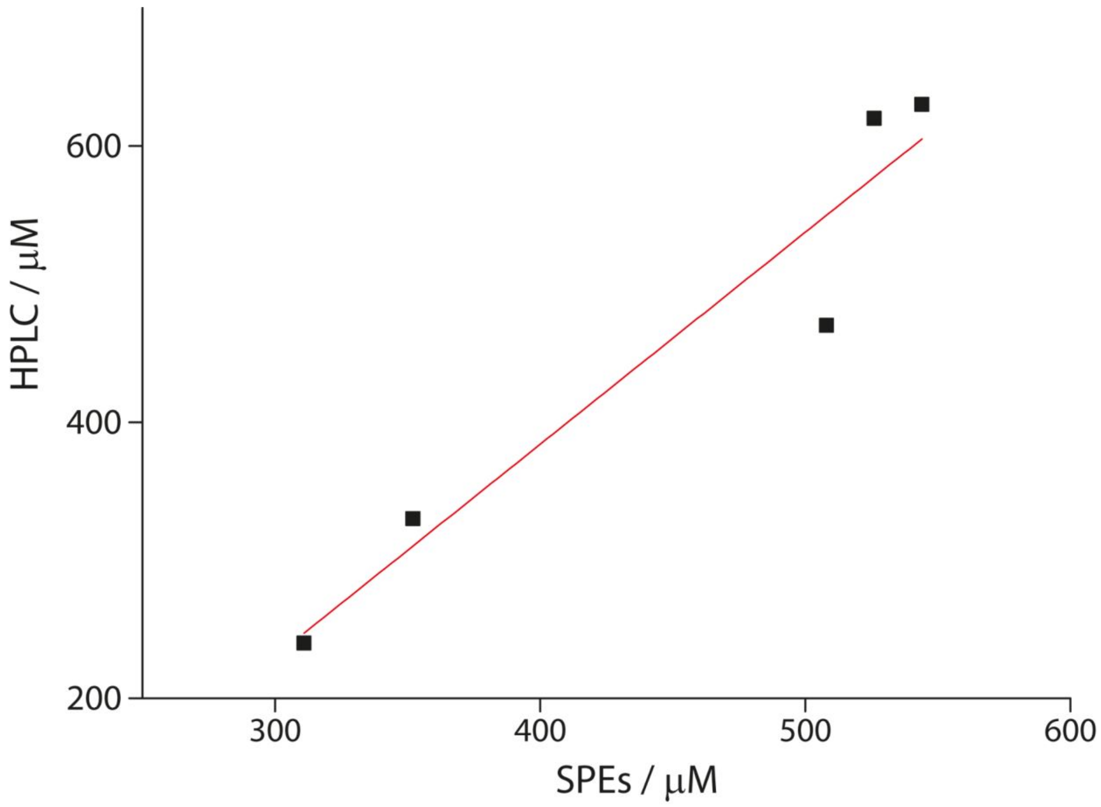

Figure S9. Correlation between uric acid concentrations measured using HPLC and the screen-printed electrochemical biosensor in real saliva samples. The red line represents the linear regression fit ($y = 1.53x - 230.50$, $R^2 = 0.960$).
